# Chronic Treatment with the IDO1 Inhibitor 1-Methyl-D-Tryptophan Minimizes the Behavioural and Biochemical Abnormalities Induced by Unpredictable Chronic Mild Stress in Mice - Comparison with Fluoxetine

**DOI:** 10.1371/journal.pone.0164337

**Published:** 2016-11-09

**Authors:** Anthony Laugeray, Jean-Marie Launay, Jacques Callebert, Oguz Mutlu, Gilles J. Guillemin, Catherine Belzung, Pascal R. Barone

**Affiliations:** 1 Molecular and Experimental Immunology and Neurogenetics – UMR7355, CNRS - 3b Rue de Férollerie, Orléans La Source, Cedex 2, France; 2 UMRS INSERM U942, Service de Biochimie, Hôpital Lariboisière, Assistance Publique — Hôpitaux de Paris, Paris, France; 3 UMRS INSERM U930, CNRS ERL 3106, Université François Rabelais, Tours, France; 4 Department of Pharmacology, Faculty of Medicine, Kocaeli University, 41380 Kocaeli, Turkey; 5 Neuroinflammation Group, Faculty of Medicine and Health Sciences, Macquarie University, NSW, Sydney, Australia; INSERM, FRANCE

## Abstract

We demonstrated that confronting mice to the Unpredictable Chronic Mild Stress (UCMS) procedure—a validated model of stress-induced depression—results in behavioural alterations and biochemical changes in the kynurenine pathway (KP), suspected to modify the glutamatergic neurotransmission through the imbalance between downstream metabolites such as 3-hydroxykynurenine, quinolinic and kynurenic acids. We showed that daily treatment with the IDO1 inhibitor 1-methyl-D-tryptophan partially rescues UCMS-induced KP alterations as does the antidepressant fluoxetine. More importantly we demonstrated that 1-methyl-D-tryptophan was able to alleviate most of the behavioural changes resulting from UCMS exposure. We also showed that both fluoxetine and 1-methyl-D-tryptophan robustly reduced peripheral levels of proinflammatory cytokines in UCMS mice suggesting that their therapeutic effects might occur through anti-inflammatory processes. KP inhibition might be involved in the positive effects of fluoxetine on mice behaviour and could be a relevant strategy to counteract depressive-like symptoms.

## Introduction

There is strong evidence that tryptophan (TRP)/kynurenine (KYN) pathway plays a key role in the link between inflammation and affective disorders [1–3]. Indoleamine-2,3-dioxygenase (IDO1; EC 1.13.11.52) is one of the two enzymes (the other is tryptphan-2,3-dioxygenase; TDO) metabolizing tryptophan (TRP) along the kynurenine pathway (KP). *In vitro*, IDO1 is induced by proinflammatory cytokines such as IFN-γ, IFN-α, IFN-β and TNF-α [4,5]. Other cytokines such as IL-12, IL-6 and IL-1 or lipopolysaccharide are also capable of inducing IDO1, although in a lesser extent [6,7]. IDO1 has been proposed to lie at the interface between chronic inflammatory disease and depression [8,9]. Reduced circulating tryptophan level and a concomitant increase in KYN have been reported in patients with chronic inflammatory diseases [10,11] and major depression for nearly two decades [5,12–14]. Moreover, reversal of the kynurenine pathway may improve depression in HIV-infected patients [15]. Interestingly, increased IDO1 activity has been found to be positively correlated with the severity of depressive scores [5,16], due to high level of circulating proinflammatory cytokines which is a common feature in these patients [14,17,18].

In support of a major role of this enzyme in stress-induced disorders, preclinical studies have shown that IDO1-mediated activation of the KP by systemic immune challenges resulted in both anxiety and depressive-like symptoms in mice [19]. More importantly, the IDO1 inhibitor 1-methyltryptophan (1MT) is able to reverse this effect [19–21]. These data strongly suggest that blocking IDO1 could be useful to alleviate stress-induced behaviours. However, the precise roles of KP activation in depressive states and other stress-related disorders remains under-explored, in particular the way by which it may promote neuropsychiatric symptoms.

In this realm, preclinical and clinical studies have brought some important clues on how activated KP could generate mood changes. Indeed, it has been shown that downstream metabolites such as quinolinic (QUIN) and kynurenic (KYNA) acids can modulate stress-related behaviours in rodents by respectively enhancing and reducing extracellular levels of glutamate [22,23]. Such an assumption is of value given the central role of the glutamatergic system in mood-disorders [24–26]. QUIN also induces cell damages through excitotoxic lesion and oxidative stress [27]. Other KP metabolites such as 3-hydroxykynurenine (3HK) have also been shown to be important generators of free radicals mediating oxidative stress [28]. Therefore, accumulation of such compounds into the brain may disturb cellular and molecular processes leading to altered neuroplasticity and may also possibly lead to neurodegeneration [29]. In accordance with such an assumption, we brought evidence in a previous study that exposing mice to an unpredictable chronic mild stress (UCMS) regimen resulted in a shift between QUIN and KYNA pathways in corticolimbic structures [30]. Particularly in the brain, the balance seems to be diverted towards QUIN pathway in the amygdala and striatum and towards KYNA pathway in the cingulate cortex. However, an important question remained unanswered: were these changes causally related to UCMS-induced emotional changes? If so, KP inhibition should reverse, even at least partially, UCMS-induced emotional changes. In a recent study, it was shown that mice submitted to social defeat stress exhibited elevated blood levels of pro-inflammatory cytokines and brain levels of kynurenine metabolites in the amygdala and the hippocampus, and that IDO1 inhibition abolished stress-induced kynurenine pathway abnormalities [31].

Our latest study examined biochemical and behavioural effects of a chronic treatment with the IDO1 blocker 1MT on UCMS-induced alterations. We also compared 1MT effects with the classical antidepressant fluoxetine, well known to reverse emotional abnormalities caused by the chronic stress [32]. Biochemical analyses included measurements of TRP-KYN pathway metabolites KYN, 3HK, KYNA and QUIN. KP metabolites were also measured in three corticolimbic structures in which KP changes were reported in our previous study [30]: cingulate cortex (CC), amygdala (AMY), and striatum (STR).The concentration of these compounds was also determined in lungs in order to evaluate the peripheral activity of the KP and because IDO1 is strongly expressed in this tissue while TDO is not [33]. As such, changes in KP metabolite levels were likely to result from IDO-dependent mechanisms. In addition, we previously showed that IDO activity in lungs is related to some UCMS-induced behavioural alterations [34].

## Methods

### Animals

Male BALB/c mice (Centre d'Elevage Janvier, France), aged 7 weeks were used. Animals were kept in the laboratory for 2 weeks before the onset of the experiment. Animals were split into 2 groups: the non-stress (control) mice and mice subjected to UCMS. Non-stress mice were group-housed in standard laboratory cages (42x28x18cm) (4 mice/cage) while UCMS mice were housed in small individual cages (24x11x12cm) at the beginning of the UCMS until the end of the study. Non-stress mice were maintained under standard laboratory conditions (12h light: 12h dark cycle, lights on at 08:00 pm, T = 21±1°C) in a separate room. All animals received food and water *ad libitum*. All aspects of animal care and experimentation were in accordance with the European Parliament and Council Directive (2010/63/EU). Our local Ethics Committee ("Comité d'Ethique en Expérimentation Animale Val de Loire.") approved all animal care and use for this study.

### Drugs

Fluoxetine hydrochloride (FLX) was obtained from Sequoia Research Products Ltd (UK). The IDO inhibitor 1-methyl-D-tryptophan (1-MT) was purchased from Sigma-Aldrich and dissolved in saline (NaCl 0.9%) containing 10% dimethyl sulfoxide. Vehicle, FLX (15mg/kg/day) and 1-MT (70mg/kg/day) were administered intraperitoneally (i.p.), based on previous experiments [35] and literature data [36]. Both drugs were injected every day (1pm) from the second week of UCMS till the end of the experiment.

### Unpredictable chronic mild stress

**Fig 1:**
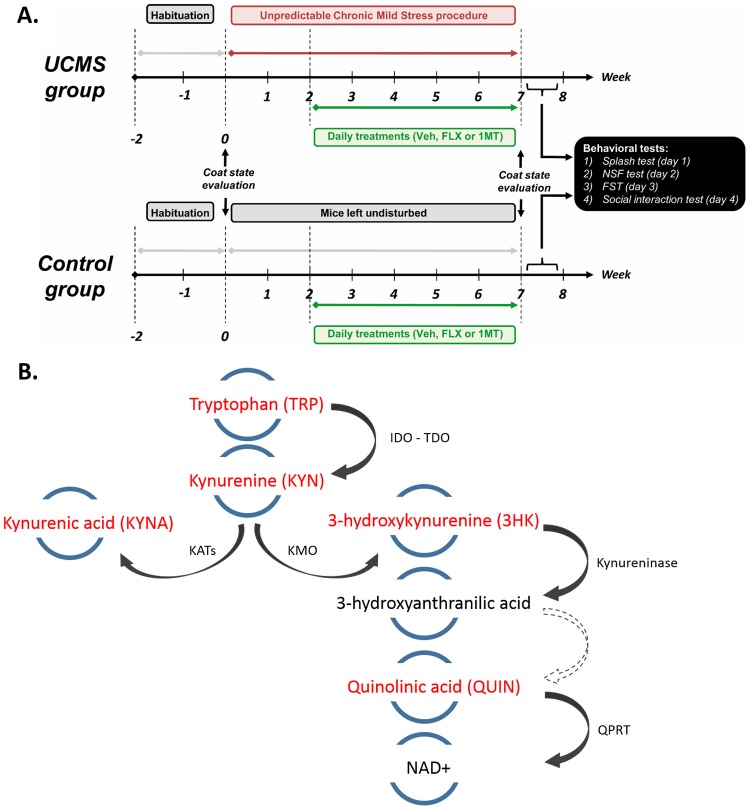
Experimental design of the study. **A.** On their arrival, mice were kept undisturbed in the laboratory for two weeks before the beginning of the experiments. A 7-week UCMS procedure was then conducted on half of the mice. The first two weeks of stress were drug-free; pharmacological treatment started from the beginning of the third week and continued until the end of the behavioural testing period. Vehicle (Veh), fluoxetine (FLX) and 1-methyltryptophan (1-MT) were administered i.p. once a day at 1 pm. Experimental groups were constituted as follow: control/veh, UCMS/vehicle, UCMS/FLX and UCMS/1-MT. Each group consisted of 9–12 animals. Fur state was assessed before and after the stress regimen. The day after the last coat state evaluation, behavioural tests were carried out as follow: novelty suppressed feeding on day 1, splash test on day 2 and resident/intruder (R/I) test on day 3. **B.** Simplified graphical representation of the kynurenine pathway; Compounds in red are those measured in the study.

Chronic stress is a major risk factor for the development of clinical depression. The unpredictable chronic mild stress (UCMS) protocol is an established, translationally relevant model for inducing behavioural symptoms commonly associated with clinical depression (anxiety, altered grooming behaviour in rodents), physiological (hypercortisolemia, hypertension) and neurological (anhedonia, learned helplessness) changes that are clinically associated with depression [37]. As such, the UCMS protocol offers many advantages over acute stress protocols or protocols using more severe stressors. UCMS is a variant of the chronic mild stress procedure described by Willner in rats [38]. UCMS regimen was applied as described in our previous study with slight changes [30]. Our protocol involved randomized, daily exposures to distinct stressors such as damp bedding, removal of bedding, cage tilt, alteration of light/dark cycles, social stresses, shallow water baths, and predator sounds/smells. Mice were subjected 3–4 hrs per day to these mild stressors for 7 weeks (see S2 Fig for the detailed schedule). None of the stressors involved water or food deprivation. In order to prevent habituation and maintain the aspect of unpredictability, the number of stressors and their duration changed every day (S2 Fig). The experimental design of the study can be appreciated on Fig 1A.This paradigm has good face and predictive validity, making such a procedure the most valuable tool to study depressive symptoms resulting from chronic stress exposure [37].

### Behavioural analyses

#### Coat state

Coat state was used as an index of depressive-like state and was assessed before and after chronic stress procedure. This index has been pharmacologically validated in previous studies [39–41]. The total score of the coat state resulted from the sum of scores obtained from 5 different body parts: head, neck, dorsal coat, ventral coat, and hindpaws. For each body area, a score of 0 was given for a well-groomed coat and 1 for an unkempt coat. Thus the more elevated the total score the nastier the fur. Dirty state is characterized by a fluffy, greasy, less dense coat or piloerection. The evaluation was carried out by 2 observers who were completely unaware of the treatment condition.

#### Splash test

The splash test was used to evaluate mice motivation to initiate self-care grooming. Several reports showed that UCMS decreased grooming behaviours [32,40,42,43], a form of motivational behaviour considered to be similar to some symptoms observed in depressed patients such as apathy [44]. Moreover, it was previously shown that UCMS-induced grooming perturbation is associated with hedonic reactivity in the sucrose preference test and increased immobility in the force swim test [45,46]. The splash test consisted of splashing a 10% sucrose solution on the mouse’s dorsal coat in its home cage. Because of its viscosity, the sucrose solution dirties the mouse fur and the animals initiate self-care grooming behaviours. Immediately after having been splashed, the behaviour of the mice was videotaped over 5 min and the videos were later analysed by a single observer who was unaware of the treatment. The test was performed in a different room. Two parameters were measured: latency to groom and, time spent grooming. Grooming bouts included nose/face grooming (strokes along the snout), head washing (semicircular movements over the top of the head and behind the ears) and body grooming (body fur licking) [47].

#### Novelty-suppressed feeding test

This test induced a conflict between the drive to eat and the fear of exploring new environments. The procedure used in this study was a modified version of that used by Santarelli *et al*. [40]. The apparatus consisted of a wooden box (33x33x30cm) illuminated by an indirect light (10lx). The floor was made of black Plexiglas. Food was removed from the mice cages 12h before the test. At the time of testing, a single pellet of regular chow was placed at the centre of the box. Mice were placed in a corner of the box and the latency to chew the pellet was recorded for 3min. The use of the video-tracking system Ethovision XT7 software (Noldus^®^) allowed a more detailed analysis of mice behaviour. Therefore, 2 more parameters were scored: total distance travelled and immobility time. Given that chronic stress protocols are known to induce unspecific changes, in particular in locomotion parameters, that would be triggered in response to acute stressors inherent to testing conditions [48].

#### Resident/Intruder test

This paradigm investigates aggressive behaviours, frequently associated not only with depression (about 30%–40% of patients) [49] but also with generalized anxiety [50]. The resident/intruder (R/I) test was modified from previously described protocols [41,51]. Control mice were single-housed 24h before testing in new cages (24x11x12cm). UCMS mice were also placed in new cages 24h prior to testing. All mice were tested against a non-aggressive intruder (male A/J mice). The opponent was placed into the home cage of the resident so that mice were in opposite corners. The dyadic encounter lasted for 5min and was videotaped to allow subsequent behavioural analysis by a single observer, blind to the treatments. All behavioural scoring focused on the resident mouse. Duration of aggressive encounters was scored from videotapes. Behaviours interpreted as aggressive were offensive behaviours such as vigorous sniffing of the opponent, full-blooded adjacent lying, tail rattling, chasing, biting and fighting.

### Tissue sampling

#### Brain tissue

Brain structures were collected 1 day after the end of the behavioural testing period and were then microdissected. Brains were rapidly removed from CO_2_-killed mice and placed in ice-cold slurry of 0.9% NaCl. Rostro-caudal sections (2mm) were quickly obtained on a brain tissue blocker. Four consecutive sections from Bregma+2.4 to -3.1 were then microdissected [52]. The cingulate cortex (CC) was dissected from the first 2 sections and included prelimbic and cingulate cortices. Dorsal and ventral parts of the striatum (STR) were obtained from the second slice. The amygdala (AMY) was obtained from the third section. All samples were immediately frozen and stored at -80°C.

#### Peripheral tissues

Lungs were removed just after the brain and immediately frozen and stored at -80°C. This tissue was chosen because IDO1 is highly expressed while TDO is not [33]. Such a strategy enabled us to specifically evaluate the effect of pharmacological treatments on IDO1.

### Biochemical measurements of TRP metabolites

A simple scheme of the kynurenine pathway highlighting the metabolites measured in the study is provided in Fig 1B. TRP was measured using HPLC, as described by Kema *et al*. [53]. Metabolites of the kynurenine pathway: KYN, KYNA and 3HK were measured using HPLC, as described by Fujigaki *et al* [54]. QUIN was measured in tissue homogenates using mass-fragmentography as previously reported [55,56].

### Measurements of peripheral cytokine levels

Cytokines (TNF-α, IL1-β, IL-6 and IFN-γ) were measured in the same lung homogenates as TRP metabolites by ELISA kits (MTA00B, SMLB00C, M6000B, DY485 respectively, R & D systems, Minneapolis, USA) according to the manufacturer's instructions.

### Statistical analyses

As data did not fit the homogeny of variance and normality, non-parametric procedures were used to analyse the results. These tests were especially adapted to the statistical analysis of small samples (*n*<30) as is the case in this study. General comparison among groups was made by Kruskal-Wallis ANOVA. When this test was significant, the Mann-Whitney U test was used to compare one group to another. As multiple comparisons were performed, we used the Bonferroni correction to avoid spurious positives. Consequently, all reported p values are corrected (= p×3). Spearman's rank correlations were calculated to describe associations between variables of interest. All data was analysed with Statistica 8 software.

## Results

### Behavioural data

**Fig 2:**
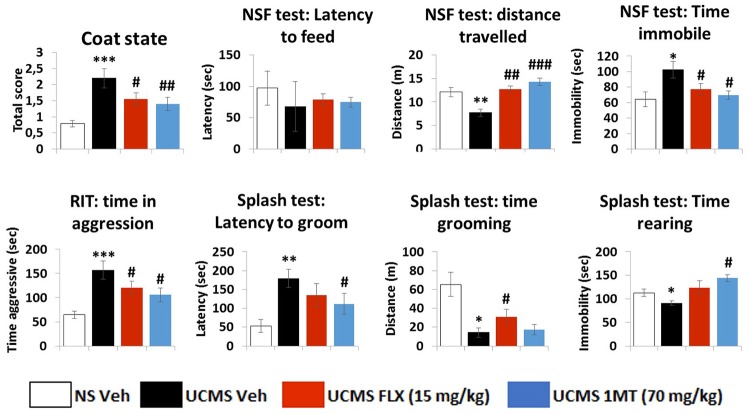
Behavioural effects of the stress regimen and chronic treatment with fluoxetine or 1-methyltryptophan. UCMS and treatments (fluoxetine: 15mg/kg and 1MT: 70mg/kg) effect on coat state score, motivation to groom in the splash test, anxiety-like behaviours in the novelty suppression of feeding test and aggressiveness in the resident-intruder test. Data are mean ± sem. N = 9-12/group. Multiple comparisons were performed. Consequently, significant p values were corrected according to the method of Bonferroni. * p<0.05; ** p<0.01 and *** p<0.001 in comparison to vehicle-treated non stressed mice. # p<0.05; ## p<0.01 and ### p<0.001 in comparison to vehicle-treated UCMS mice.

Behavioural data is summarized in Fig 2. UCMS not only altered the coat state of stressed mice (p < 0.001) but also the behavioural response measured in the NSF test as mice displayed reduced locomotor activity (p = 0.007) and were significantly more immobile (p = 0.03) compared to non-stressed animals. However, no changes were observed regarding latency to chew the food pellet. In the resident-intruder test (RIT), UCMS mice were also shown to be more aggressive towards an intruder compared to non-stressed animals (p < 0.001). Behavioural alterations were also observed in the splash test as UCMS displayed increased latency to groom (p = 0.008) concomitant to a reduced time spent grooming (p = 0.02). In line with these changes, UCMS mice spent significantly less time rearing (p = 0.03). Interestingly, both the chronic treatment with the IDO1 inhibitor 1MT and the antidepressant fluoxetine partially reversed the aversive effects of the UCMS on the coat state (p = 0.009 and p = 0.04 respectively), on the distance travelled (p < 0.001 and p = 0.006 respectively) and the time spent immobile (p = 0.03 and p = 0.04 respectively) during the NSF test. Similarly, both 1MT and fluoxetine significantly rescued mice behaviour in the RIT (p = 0.04 and p = 0.047 respectively). The two compounds were also effective in reducing UCMS-induced behavioural alterations in the splash test but this beneficial effect was different depending on the behavioural outcome: 1MT reversed UCMS-induced increase in latency to groom and reduced time rearing (p = 0.03 for both) whereas FLX was ineffective. And FLX significantly counteracted the effect of UCMS on time spent grooming (p = 0.04) while 1MT did not.

Taken together, the results indicate that daily treatment with the IDO1 inhibitor 1MT and the antidepressant fluoxetine restored behavioural changes induced by the UCMS regimen.

### Biochemical data: kynurenine pathway changes

#### In the periphery: lungs (Fig 3A)

IDO1 activity, estimated by the KYN/TRP ratio, tended to be increased in response to UCMS but the difference did not reach statistical significance (p = 0.15). Interestingly, both 3HK and QUIN were significantly raised in UCMS mice (p < 0.001 and p = 0.02 respectively) while KYNA remained unchanged compared to controls. While neither the IDO1 activity nor the KYNA pathway were significantly affected by the stress regimen, it is interesting to note that both 1MT and fluoxetine treatment potently reduced IDO1 activity (p < 0.001 for both) and KYNA levels (p < 0.001 and p = 0.008 respectively). The 2 treatments were also able to robustly reduce 3HK (p < 0.001 for both) and QUIN (p < 0.001 for both).

**Fig 3:**
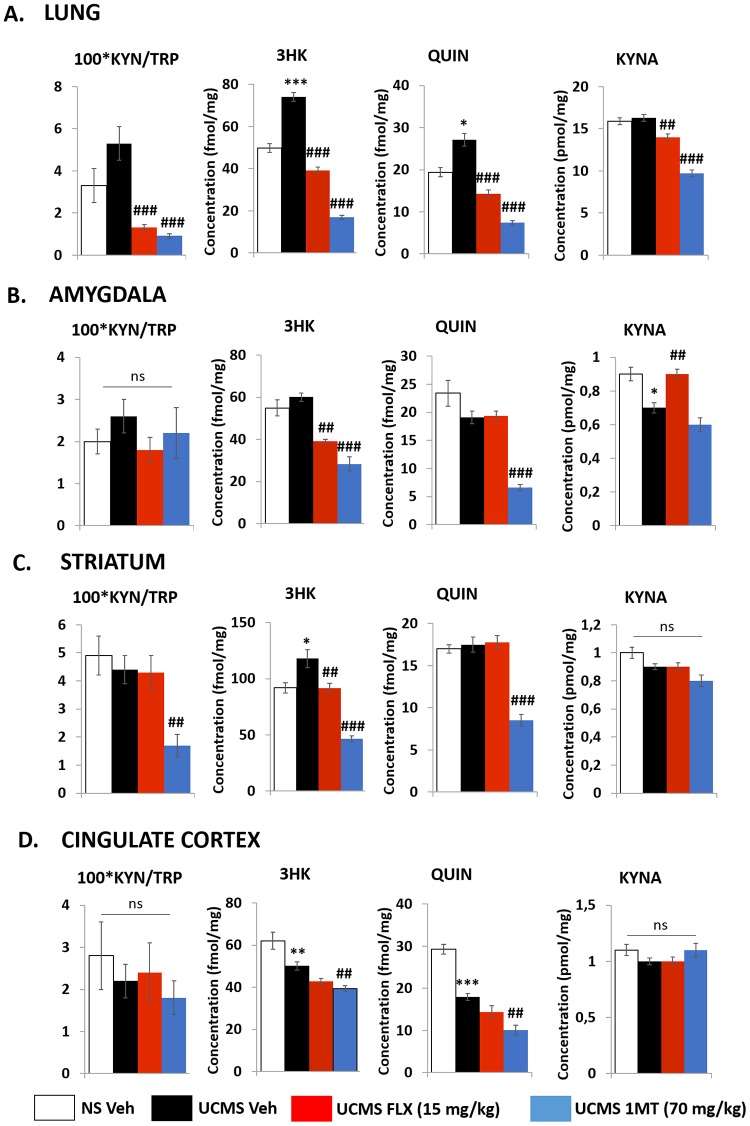
Effect of UCMS and chronic treatment with fluoxetine or 1-methyltryptophan on the kynurenine pathway. UCMS / treatment (fluoxetine: 15mg/kg and 1MT: 70mg/kg) effects on the **(A)** peripheral and **(B-D)** brain kynurenine pathway metabolites. Peripheral measurements were made in lungs while brain measurements were made in amygdala (AMY) **(B)**, striatum (STR) **(C)** and cingulate cortex (CC) **(D)**. Kynurenine (KYN), tryptophan (TRP), 3-hydroxykynurenine (3HK), quinolinic acid (QUIN) and kynurenic acid (KYNA) were measured as described in the “materials and methods” section. KYN/TRP ratio was computed as an index of IDO1 activity. Data are mean ± sem. N = 9-12/group. Multiple comparisons were performed. Consequently, significant p values were corrected according the method of Bonferroni. * p<0.05, *** p<0.001 in comparison to vehicle-treated non-stressed mice, ## p<0.01 and ### p<0.001 in comparison to vehicle-treated UCMS mice.

#### In the brain at the subcortical level

In the amygdala (Fig 3B), UCMS had no effect on the KYN/TRP ratio, 3HK and QUIN levels whereas KYNA was significantly decreased compared to non-stressed mice (p = 0.02). IDO1 activity was not modified in response to pharmacological treatments. Interestingly, both 3HK and QUIN, even if not changed in UCMS mice, were reduced in 1MT-treated UCMS mice (p < 0.001 for both) while UCMS-induced KYNA reduction was not restored by 1MT. FLX also exerted biochemical effects by restoring KYNA level to that of the non-stressed animals (p = 0.009) and by reducing 3HK level compared to vehicle-treated UCMS mice (p = 0.006). in the striatum (Fig 3C), UCMS did not modify the KYN/TRP ratio, KYNA and QUIN levels but significantly increased 3HK (p = 0.02). This alteration was completely reversed by 1MT and FLX treatments (p < 0.001 and p = 0.009 respectively). Of note, both the KYN/TRP ratio and QUIN were significantly decreased in 1MT-exposed UCMS (p = 0.008 and p < 0,001 respectively) mice while FLX did not exert any effect on these parameters.

#### In the brain at the cortical level

In the cingulate cortex (Fig 3D), neither KYNA nor the KYN/TRP ratios were affected by UCMS while 3HK and QUIN were significantly decreased (p = 0.009 and p < 0.001 respectively). Interestingly, FLX did not change concentrations of 3HK and QUIN whereas 1MT amplified the effect of UCMS on both 3HK and QUIN levels (p = 0.007 and p = 0.005 respectively). The 2 treatments were without any effect on KYNA and KYN/TRP ratio.

### Cytokine quantification in the lungs

**Fig 4:**
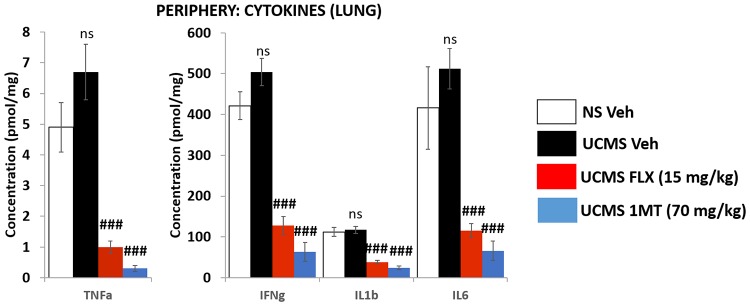
Effect of UCMS and chronic treatment with fluoxetine or 1-methyltryptophan on peripheral (LUNG) levels of TNF-α, IL1-β, IFN-γ and IL6. TNF-α, IFN-γ, IL1-β and IL6 were measured as described in the “materials and methods” section. Measurements were performed in lungs because of its high levels of IDO1 expression. Data are mean ± sem. N = 9-12/group. Multiple comparisons were performed. Consequently, significant p values were corrected according the method of Bonferroni. Ns indicate no difference compared to vehicle-treated non-stressed mice. ### p<0.001 in comparison to vehicle-treated UCMS mice.

Data is summarized in Fig 4. None of the four cytokines studied, TNF-α, IL1-β, IFN-γ and IL-6, were significantly affected by the stress regimen. However, the 2 pharmacological treatments were able to potently decrease the level of TNF-α, IL1-β, IFN-γ and IL-6 (p<0.0001 for both fluoxetine and 1MT).

## Discussion

### 1MT efficiently reduced UCMS-induced emotional alterations

The main finding of this study is clearly that the IDO1 blocker 1MT is able to prevent UCMS-induced emotional alterations, highlighting the idea that the KP is likely to be involved in the pathogenesis of stress-induced behavioural alterations. Indeed, UCMS produced its usual effect on the coat state test, the splash test and the resident-intruder test [41,51,57] that were partially prevented by 1MT treatment. With the NSF test, we did not observe any increase in the latency to chew the pellet as it is classically reported in several studies [32,40,41,58], which is possibly associated with the use of an inappropriate light level when the test was conducted. Despite this lack of effect, it is interesting to note that UCMS mice displayed other behavioural abnormalities that could be informative for the assessment of 1MT-induced behavioural effects, the main goal of the study. Therefore, UCMS mice displayed decreased locomotion in the NSF test. This result was indicative of an overall effect of chronic stress on emotional reactivity in response to novelty. This view is in line with other findings showing that chronic stress may induce unspecific changes that would be triggered in response to acute stressors inherent to testing conditions [48].

As previously mentioned, the kynurenine pathway is not only activated by the enzyme IDO1 but also by the enzyme TDO2. However, such behavioural effects of 1MT could not be explained by the inhibition of the TDO2 as 1MT has been shown to specifically act on IDO1 [59]. Nonetheless, it was recently demonstrated that TDO2 inhibition blocked chronic stress-related emotional alterations in mice [60]. Even if our results suggest that IDO1 is importantly involved in UCMS-induced emotional alterations, the present report does not preclude the possibility of an involvement of the enzyme TDO2. Such an assumption could likely explain why the beneficial effects of 1MT is only partial. Maybe the joint administration of TDO2 and IDO1 inhibitors could be of particular value to relief chronic stress-induced alterations.

Previous studies have already brought evidence for such a determinant role of 1MT in the modulation of stress-induced emotional alterations. O’Connor *et al*. showed that blocking IDO1 by 1MT could partially reverse inflammation-induced depressive-like states in mice [20,21], while recent findings by Dobos *et al*. showed that IDO1 inhibition by 1MT in centrally induced neuroinflammation in mice can prevent the development of depressive-like behaviours [61]. These findings strongly suggest that IDO1 activation is an important feature of anxiety-like and depressive-like states induced by inflammatory processes. However, not all depressive states are of inflammatory origin but also of psychological origin. In such cases, very little was known about the involvement of IDO1 and the KP in the pathophysiology of stress-induced anxiety and depression until the present study. Indeed, our results showed for the first time that KP disturbances might constitute a relevant hallmark of stress-induced emotional alterations and that blocking this activation might constitute an innovative treatment perspective for patients suffering from the psychogenic effects of chronic stress. This assumption is strengthened by work from Kiank *et al*. which showed that 1MT was able to prevent emotional alterations induced by sub-chronic stress in mice [62].

### 1MT completely reversed UCMS induced KP alterations

We demonstrated that UCMS disturbed the catabolism of TRP along the KP both at the peripheral level and in some relevant corticolimbic structures involved in the pathophysiology of depressive symptoms in humans. Our results showed the peripheral KP was diverted through the synthesis of 3HK and QUIN, which strengthened our previous results [30].

Our earlier hypothesis was that such a phenomenon might be involved in UCMS-induced behavioural deficits through an elevated supply of KYN in the brain, which could have disturbing effects on cerebral homeostatic processes through local changes in neuroactive metabolites such as 3HK and QUIN. This hypothesis was in agreement with the fact that an exogenous supply of KYN dose-dependently affects emotion-based behaviours in naïve mice [21]. These results implied that increasing systemic KYN levels may result in an increased synthesis of neuroactive KP metabolites (such as 3HK and/or QUIN) in the brain that are likely to be the real effective mediators as suggested in several reports [34,63]. The present study strengthened this assumption as UCMS significantly increased KYN metabolism along the 3HK pathway in the periphery despite a lack of effect on IDO1 activity. While the effect of UCMS in the KYN/TRP ratio did not reach statistical significance even if the trend was upwards, this discrepancy can be explained by the inherent variability between UCMS-exposed individuals. This possibility is in agreement with our previous work showing that UCMS mice with an elevated lung KYN/TRP ratio also displayed high emotion-based behaviours in the elevated plus maze and the forced swim tests [34]. Additionally, in the present study we showed that IDO1 activity (KYN/TRP ratio) in lungs was positively correlated to local levels of IL-1β, IFN-γ and IL-6 (S1 Fig) only in the mice exposed to UCMS. These results confirmed our hypothesis about the link between UCMS-induced IDO1 activity and inter individual variability. Taken together, these results highlight the need to further investigate the link between resilience to stress and KP changes.

The fact that daily treatment with 1MT completely reversed UCMS-induced increase in 3HK and QUIN in lungs on the one hand, and blocked UCMS-induced emotional changes on the other hand, strongly strengthened the link between peripheral KP changes and stress-related symptoms. We also demonstrated in the brain that most UCMS-induced KP changes were reversed by 1MT treatment suggesting that behavioural effects might be the consequence of such changes. Interestingly, we did not observe any effects of UCMS on the KYN/TRP ratio irrespective of the structure we studied. This was in agreement with our initial study [30]. Nevertheless we showed KYN to be preferentially metabolized along the 3HK pathway in the striatum, as reflected by the increase in 3HK levels. Again, this result was in agreement with our previous study which also showed an increase in3HK synthesis in this structure [30]. Whereas KYNA was decreased by UCMS in our previous report, it was not significantly altered in the present study even though KYNA tended decrease. In the amygdala, the current results were also partially in accordance with the observations in our initial study. Only KYNA was reduced in the present work whereas, previously, we showed that UCMS reduced KYNA concomitantly to increase 3HK [30]. While we did not obtain exactly the same results, our present data suggested that UCMS may shift the balance between 3HK and KYNA in favour of the former, being potentially responsible for 3HK-related local damages [28]. We also demonstrated that daily treatment with the IDO1 inhibitor 1MT only partially abrogated these pathological effects of UCMS, reinstating the original equilibrium between the 2 arms of the KP within the striatum but not in the amygdala where UCMS-induced changes in KYNA were not restored by 1MT. These results suggest that the KP could be differentially regulated depending on the brain structure and subsequently that various cellular processes might be at work. Notably, we observed that UCMS reduced the 3HK/QUIN pathway in the cingulate cortex, especially QUIN levels and that 1MT accentuated this phenomenon. This result was in line with data reported in our previous work and confirmed that the KP was differentially regulated depending on the brain structure. Such a mechanism was quite surprising and we did not have any particular explanations about the origin of this phenomenon. These results need to be studied more precisely to fully unravel the underlying processes. The enzyme TDO, known to be expressed in the cortical structures of mice and activated by chronic stress [60], may also be involved in such a phenomenon. In addition, it is important to keep in mind that plasma level of KP metabolites was not determined in the present study. Performing such measurements could be of value to support our hypothesis on the role of the peripheral KP in influencing brain KYN metabolism.

Collectively, biochemical data showed differential effects of 1MT treatment depending on the tissue studied: indeed, 1MT potently reduced the 2 branches of the KP at the peripheral level (lung). This result is consistent with a reduced IDO1 activity, expected to be associated with a decrease of both branches downstream. In the brain, only the 3HK-QUIN pathway was affected at the cerebral level. From these changes we can assume that beneficial effects of 1MT were essentially peripheral and that cerebral KP changes might be the consequence of a reduced supply of 3HK into the brain, leading to low levels of 3HK and QUIN. The fact that KYNA and KYN/TRP remained unchanged in response to 1MT treatment was in agreement with such hypothesis.

### Beneficial effects of fluoxetine on UCMS-induced changes might be due to KP modulation

As expected, our data indicated that chronic treatment with FLX—a clinically proven antidepressant—successfully reversed UCMS-induced alterations in the coat state, NSF, resident-intruder and splash tests. These results were in agreement with previous studies from our laboratory [57,64] and other groups [65]. At both the peripheral and cerebral levels, FLX potently rescued UCMS-induced effects on the KP. Two distinct mechanisms could explain such effect. First, FLX could block KP alterations through its action on the enzyme TDO, the other TRP to KYN converting enzyme. Indeed, Bano et *al*. have demonstrated that acute administration of FLX could efficiently decrease the activity of TDO in rats by inhibiting the binding between the apo-enzyme form of TDO and its co-factor [66,67]. Similar effects were shown after an acute injection of FLX in both male and female rats at 10 and 30 mg/kg doses [68]. Very recently, it was shown that inhibiting TDO promoted antidepressant activity in a mice model of chronic stress [60]. Therefore, such an inhibitory effect of FLX might constitute a key mechanism by which this molecule could reverse KP changes induced by UCMS.

Another way for FLX to exert such effect is by reversing changes in plasmatic glucocorticoids. Indeed, a chronic treatment with FLX can restore HPA axis function in UCMS mice, the consequence of which is to reinstate physiological levels of circulating corticosterone [64]. Corticosterone, being a determinant activator of the KP through TDO, a FLX-mediated diminution of corticosterone levels could reverse KP changes observed in this study.

Our results also showed that chronic treatment with FLX was able to prevent cerebral alterations of the KP metabolites in a structure dependent manner: UCMS-induced changes are restored at the subcortical level (in AMY and STR) but not at the cortical level (CC) where UCMS effect was potentiated. Such findings are in line with the recent work by Kocki *et al*. showing that in primary astroglial cultures, fluoxetine was able to diminish the level of 3-HK by down-regulating the expression of KMO, the KYN to 3HK converting enzyme [63].

Taken together, our data indicate that fluoxetine, when chronically administered, is able to prevent both peripheral and cerebral alterations of the KP, and consequently may exert its beneficial effects through KP changes.

### Immune system might be involved in 1MT and FLX beneficial effects

Our data indicated that 1MT and FLX exerted potent anti-inflammatory effects irrespective of the UCMS effect. Indeed, UCMS did not significantly change the peripheral levels of TNF-α, IFN-γ, IL1-β and IL-6. Two reasons may explain such a result. The first one relates to abnormally high levels of cytokines detected, including in control mice, probably resulting from daily intraperitoneal injections causing elevated levels of these mediators [69]. This high level of cytokines may have masked the inflammatory effects of UCMS. The second explanation could be related to what has been shown by Hodes *et al*. i.e. that individual differences in the peripheral immune system are likely to predict and promote stress susceptibility in mice [70]. Therefore, individual variability in the immune response to UCMS may have masked the effects of stress. Further work is needed to fully understand the relationships between UCMS effects and cytokine-induced KP activation through IDO1. Interestingly, 1MT has already been demonstrated to have anti-inflammatory effects [71]. Indeed, in this study the authors showed that 1MT treatment decreased inflammation processes in the lungs after Toxoplasma gondii infection, and resulted in low levels of IL1-β and IL-6 mRNA expression [71]. Given that previous studies have shown that IFN-γ and TNF-α were able to modulate the abundance of transcripts encoding several KP enzymes in vitro [72], we cannot rule out the possibility that biochemical effects of 1MT may be mediated, at least for a part, by the local reduction of proinflammatory cytokines. This hypothesis will be further addressed in our future work.

As for 1MT, FLX can also have an immunomodulatory effects resulting in a reduction of pro-inflammatory cytokine levels [73,74]. Our results were in agreement with this as FLX potently decreased the level of the 4 pro-inflammatory cytokines measured in this study at the peripheral level (lungs). As pro-inflammatory cytokines are strong KP modulators, the reversal of KP alterations might likely be the result of the anti-inflammatory effect of FLX.

In conclusion, the present study has identified IDO1 as a relevant mediator of chronic stress-induced emotional changes. We also demonstrated that chronic stress-induced emotional changes are associated with alterations in TRP metabolism along the KP resulting in higher production of neurotoxic compounds such as 3HK and QUIN. Considering their neuroactive / neurotoxic potential, changes in 3HK and QUIN levels are likely to be the effective mechanisms underlying behavioural effects of UCMS as suggested in several reports [63,75]. In addition, we assume that the beneficial effects of FLX could be mediated, at least for a part, by preventing KP alterations. But more importantly, our findings indicate that blocking IDO1 pharmacologically counteracts UCMS-induced behavioural changes and might consequently constitute an innovative treatment against behavioural alterations induced by chronic stress.

## Supporting Information

S1 Fig Correlation between the KYN/TRP ratio and cytokine levels in lungs in non-stressed and UCMS mice. TNF-α, IFN-γ, IL1-β and IL6 were measured as described in the “materials and methods” section. IFN-γ, IL1-β and IL6 were all positively correlated to lung KYN/TRP ratio highlighting the link between UCMS-induced increase in proinflammatory cytokines and IDO activation. No correlations were observed in non-stressed mice.
(TIF)

S2 Fig Detailed schedule of the UCMS protocol. Our protocol consisted in randomized, daily exposures to distinct stressors such as damp bedding, removal of bedding, cage tilt, alteration of light/dark cycles, social stresses, shallow water bath, and predator sounds/smells. Mice were subjected 3–5 hr daily to these mild stressors for seven weeks.
(TIF)

## Abbreviations

UCMS: unpredictable chronic mild stress
TRP: tryptophan
KYN: kynurenine
KP: kynurenine pathway
K/T ratio: kynurenine to tryptophan ratio
3HK: 3-hydroxykynurenine
QUIN: quinolinic acid
KYNA: kynurenic acid, IDO1, indoleamine-2,3-dioxygenase
TDO: tryptophan-2,3-dioxygenase
IFN: interferon
CC: cingulate cortex
STR: striatum
AMY: amygdala
HPLC: high performance liquid chromatography
