# Supplementary figures and images for: Chronic Treatment with the IDO1 Inhibitor 1-Methyl-D-Tryptophan Minimizes the Behavioural and Biochemical Abnormalities Induced by Unpredictable Chronic Mild Stress in Mice - Comparison with Fluoxetine
Source: PLoS One. 2016 Nov 9;11(11):e0164337. doi: 10.1371/journal.pone.0164337 (PMC5102430; doi:10.1371/journal.pone.0164337)

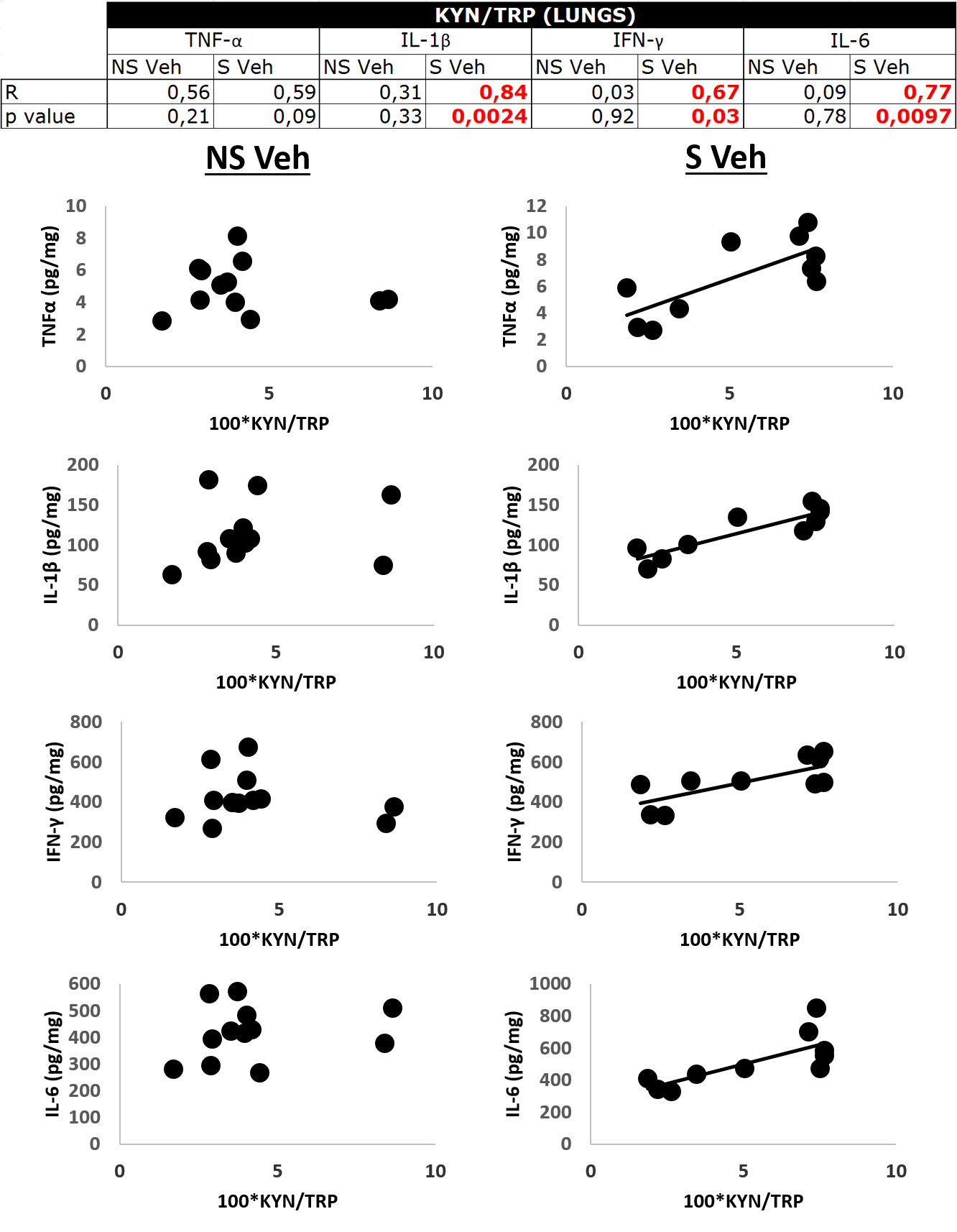

Supplement: S1 Fig — TNF-α, IFN-γ, IL1-β and IL6 were measured as described in the “materials and methods” section. IFN-γ, IL1-β and IL6 were all positively correlated to lung KYN/TRP ratio highlighting the link between UCMS-induced increase in proinflammatory cytokines and IDO activation. No correlations were observed in non-stressed mice. (TIF) [file pone.0164337.s001.tif]

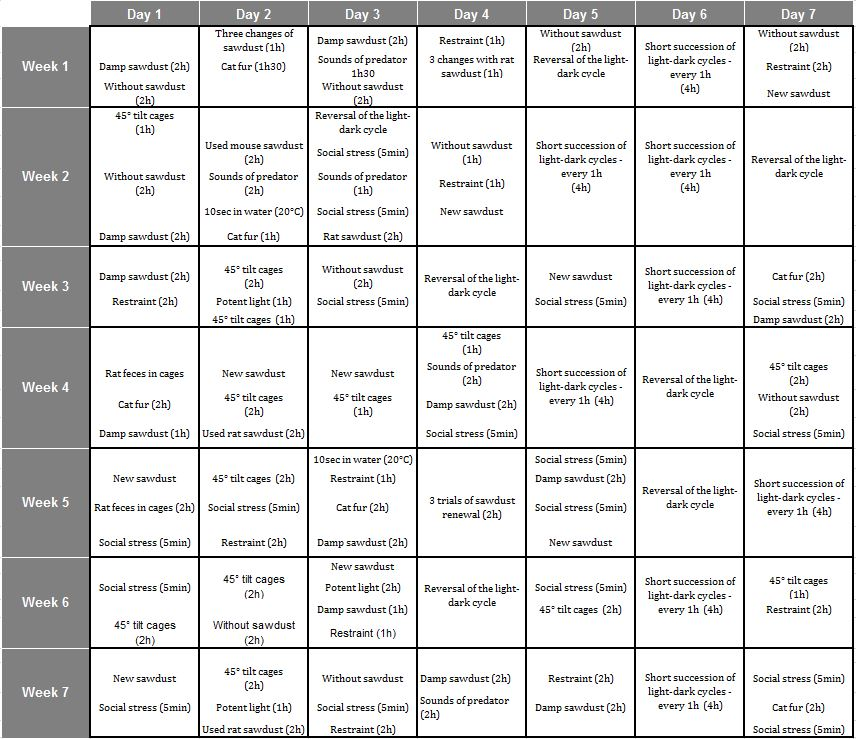

Supplement: S2 Fig — Our protocol consisted in randomized, daily exposures to distinct stressors such as damp bedding, removal of bedding, cage tilt, alteration of light/dark cycles, social stresses, shallow water bath, and predator sounds/smells. Mice were subjected 3–5 hr daily to these mild stressors for seven weeks. (TIF) [file pone.0164337.s002.tif]
